# Plant virome analysis by high-throughput sequencing: concepts and approaches

**DOI:** 10.18699/vjgb-26-35

**Published:** 2026-04

**Authors:** D.D. Belkina, S.V. Vinogradova

**Affiliations:** Federal Research Centre “Fundamentals of Biotechnology” of the Russian Academy of Sciences, Moscow, Russia; Federal Research Centre “Fundamentals of Biotechnology” of the Russian Academy of Sciences, Moscow, Russia

**Keywords:** metagenomics, high-throughput sequencing, viruses, plant virome, bioinformatics, метагеномика, высокопроизводительное секвенирование, вирусы, виром растений, биоинформатика

## Abstract

The metagenomic approach based on high-throughput sequencing is becoming increasingly prevalent for the detection of viral infections in plants. This method allows us to study the species composition of viruses associated with the plant, including novel species, describe their population genetic structure, and develop genetic test systems for routine diagnostics. A metagenomic approach to phytosanitary monitoring can help to determine the cause of unknown plant diseases, which is particularly important for preventing the spread of pathogens, such as viruses. Furthermore, as it is impossible to eliminate plant viruses in field conditions, comprehensive diagnostics using high-throughput sequencing is becoming an effective tool for complying with quarantine regulations on the import of foreign material, as well as for producing high-quality local planting material. High-throughput sequencing is becoming more affordable every year, with both the instrumentation and analytical capacity improving. This review summarizes key approaches to analyzing plant virome using high-throughput sequencing. The analysis process, from sample collection to bioinformatic data processing, validation and interpretation, is described in detail. The features of sequencing platforms and the factors affecting sequencing quality, including contamination, are discussed. Three complementary approaches to processing bioinformatic data are described: mapping reads to reference viral sequences; assembling and annotating contigs; taxonomic classification of reads without assembly. The importance of carefully interpreting the results is emphasized, considering the bioinformatic analysis and the validation by molecular genetic methods. This review will be useful for both researchers and specialists who have no experience with high-throughput sequencing, and those who have used this method for other applications.

## Abstract (ru)

В последние годы метагеномный подход, основанный на высокопроизводительном секвенировании, находит все большее применение в диагностике вирусных инфекций растений. Этот метод позволяет изучить видовой состав вирусов, ассоциированных с исследуемым растением, и в том числе обнаружить ранее не описанные виды, охарактеризовать их популяционно-генетическую структуру и разработать генетические тест-системы для рутинной диагностики. Проведение фитосанитарного мониторинга с использованием метагеномного подхода может способствовать определению этиологии неизвестных заболеваний растений, что особенно важно для предотвращения распространения таких патогенов, как вирусы. Кроме того, с учетом невозможности элиминации вирусов растений в полевых условиях комплексная диагностика с помощью высокопроизводительного секвенирования становится эффективным инструментом как в целях соблюдения карантинного законодательства при ввозе импортного материала, так и для получения отечественного посадочного материала высоких категорий. При этом с каждым годом высокопроизводительное секвенирование становится более доступным: расширяется как приборно-техническая, так и аналитическая база. В настоящем обзоре систематизированы ключевые подходы к анализу вирома растений с помощью высокопроизводительного секвенирования. В основной части статьи описаны этапы проведения анализа: от сбора образцов до биоинформатической обработки данных, ее валидации и интерпретации. Подробно рассмотрены особенности современных платформ секвенирования и факторы, влияющие на качество чтения, в том числе контаминация. Охарактеризованы три взаимодополняющих подхода для обработки биоинформатических данных: картирование чтений на референсные последовательности вирусов; сборка и аннотация контигов; таксономическая классификация чтений без дополнительной сборки. Особое внимание уделено необходимости тщательной интерпретации результатов с учетом как биоинформатического анализа, так и валидации идентификации молекулярно-генетическими методами. Обзор будет полезен как для исследователей и специалистов, не имеющих опыта работы с высокопроизводительным секвенированием, так и для тех, кто использовал этот инструмент для выполнения других задач.

## Introduction

Plant viral diseases cause substantial losses in crop production worldwide. In the context of globalization and climate change, the spread of plant viruses can lead to devastating epidemics, posing a serious threat to global food security (Jones, 2021). For instance, yield losses in tomato and pepper crops due to infection with the tomato mosaic virus (ToMV) are estimated at 25–70 % (Panno et al., 2021), whereas a reduction in grape yield caused by grapevine leafroll disease associated with viruses of the family Closteroviridae can reach 50 % (Atallah et al., 2012). In this regard, monitoring, early diagnostics and adherence to quarantine measures play a crucial role in limiting the spread of viral infections.

Currently, the identification of plant viruses is carried out mainly by serological and molecular methods, which have become widely used due to their assay speed, high sensitivity and specificity. However, these approaches enable the detection of only those virus species for which corresponding test systems have been developed (Maina et al., 2024).

Prior to the advent of metagenomics, knowledge of plant viruses was restricted to a few hundred species (Pappas et al., 2021), and consequently, the role of viruses in the microbiome of agricultural plants remained underestimated. The metagenomic approach allows us to obtain an in-depth understanding of all viruses of a plant, or its virome. As of 2025, the International Committee on Taxonomy of Viruses (ICTV) has registered 2,598 plant viruses and viroids, including 206 added in the past year (Rubino et al., 2025). Virome sequencing provides unique information on viral genome sequences, which can be used to conduct population studies and trace pathways of virus spreading, asso-ciate specific strains with symptom expression, and develop specific test systems for their rapid detection.

Plant virome analysis using high-throughput sequencing is a rapidly evolving method. Each year, new recommendations, protocol optimizations, and specialized dataprocessing tools are published. Although this analysis has not yet become a routine diagnostic method for plant viral infections, its fundamental principles have already been established

This review systematizes key approaches to plant virome analysis using high-throughput sequencing. It will be useful for both researchers who are new to high-throughput sequencing and those who have used this technology for other applications

## General scheme of plant virome analysis

**Fig. 1.**
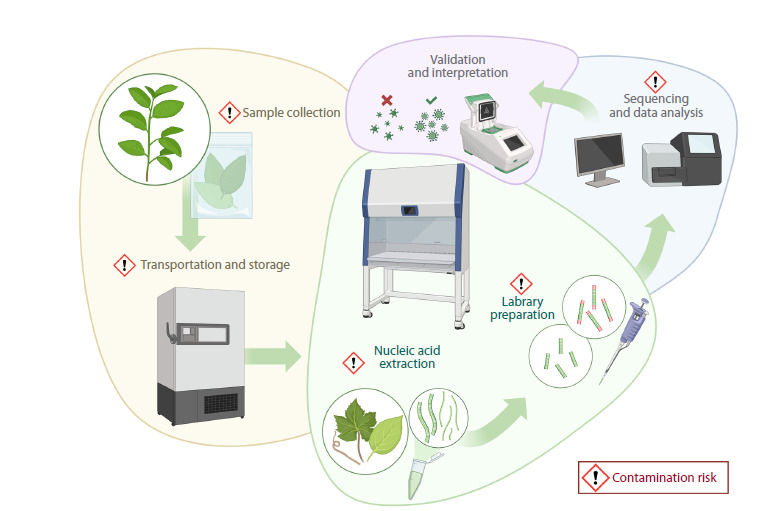
The main stages of plant virus analysis.

Structurally, virome analysis can be divided into two main stages. The first stage includes sample collection, material preparation, and sequencing (Fig. 1). This part of the analysis is crucial. If the material for sequencing is of poor quality, obtaining reliable results at subsequent steps becomes impossible. When collecting samples, it is important to consider that viruses are unevenly distributed in plant tissues, therefore, multiple fragments should be collected from each individual plant. This is particularly important for perennial plants. After collection, the samples should be transported to the laboratory as quickly as possible to minimize the risk of nucleic acid degradation, for instance, due to exposure to direct sunlight

The second stage of plant virome analysis involves computer processing of sequencing data using bioinformatics tools, followed by validation and interpretation of the results. In contrast to the steps of the first stage, bioinformatics data processing can be repeated at any time to correct potential errors, apply additional analytical methods, or double-check the results of other studies. Therefore, it is extremely important to avoid errors during sampling, sample preparation, and library sequencing. For reliable virus identification, the results of bioinformatics analysis must be validated using molecular genetic methods

## Initial material for analysis

Plant viruses differ from other pathogens in the diversity of their genome types. They can use either DNA or RNA molecules as carriers of genetic information, and these molecules may be single-stranded or double-stranded, linear or circular (Lefeuvre et al., 2019). Single-stranded RNA viruses are subdivided into those with positive-sense genomes, in which the genomic RNA corresponds to mRNA, and those with negative-sense genomes, in which the genomic RNA is complementary to mRNA. Given this genomic diversity, identifying all plant-infecting viruses using a single approach is challenging (Fitzpatrick et al., 2021). Below, we review various approaches to plant virome analysis depending on the type of templateused

Sequencing of total RNA extracted from plant tissues is a relatively simple, reliable, and widely used method for virome analysis (Lee et al., 2020; Nabeshima, Abe, 2021; Vinogradova et al., 2023). This approach enables the detection of not only RNA viruses but also transcripts of DNA viruses (Pecman et al., 2017; Cobbin et al., 2021), and viroids, making it optimal for diagnostics of viruses with different types of genomic nucleic acids.

However, the sensitivity of this method can be significantly reduced by the co-extraction of abundant host plant RNA during sample preparation (Maliogka et al., 2018). As a result, the detection of low-titer viruses becomes difficult (Roossinck et al., 2015). This problem can be mitigated by enriching the sample for virus-associated nucleic acids (Gaafar, Ziebell, 2020). Commonly employed strategies include the depletion of ribosomal and transfer RNAs or the enrichment of polyadenylated transcripts. The former method is generally preferable because not all groups of viruses have a polyadenylated 3′ end.

Another approach for plant virome analysis is sequencing virion-associated nucleic acids (Filloux et al., 2015). This approach has the advantage of targeting exclusively viral sequences, which makes it possible to analyze both RNA and DNA viruses, including those present at low titers (Moubset et al., 2022). However, it is limited to the detection of viruses with a capsid, which is absent in satellite viruses and viroids, and it requires more effort than the extraction of total RNA (Maliogka et al., 2018).

The next approach involves the analysis of short interfering RNAs (siRNAs), which are produced in plant cells as a result of the immune response to viral infection. These molecules represent fragments of viral genomes cleaved by plant enzymes, 21–24 nucleotides in length (Vivek et al., 2020; Zhuravlyov et al., 2022).

While siRNA sequencing can detect both RNA and DNA viruses, the short length of these molecules complicates the de novo assembly of complete viral genomes (Pecman et al., 2017; Maliogka et al., 2018; Turco et al., 2018).

Many viruses produce double-stranded RNA (dsRNA) as a replication intermediate, which can also be used in plant virome studies. Sequencing of dsRNA enables the identification of not only dsRNA viruses but also most single-stranded RNA viruses, viroids, and some DNA viruses (Gallo et al., 2021; Fall et al., 2025). Nevertheless, this method is less efficient for detecting DNA viruses and typically yields positive results only at high levels of viral transcript expression (Gaafar, Ziebell, 2020).

## Sequencing platforms

All metagenomic studies are based on high-throughput sequencing (HTS) of nucleic acids. This group of methods includes second- and third-generation sequencing that replaced Sanger sequencing. While Sanger sequencing remains the gold standard for reading sequences up to 1,000 nucleotides in length, its throughput is insufficient for metagenomic applications (Crossley et al., 2020).

Second-generation sequencing, also referred to as nextgeneration sequencing (NGS) or massively parallel sequencing, generates large volumes of short reads, typically several hundred nucleotides in length. This capability enables the rapid and comprehensive sequencing of all sequences in a sample. However, the short read length complicates the assembly of the complete viral genome during bioinformatics analysis (Maina et al., 2024).

The leader in the NGS market is Illumina (USA), whose platforms are characterized by high throughput and low error rates (Maina et al., 2024). In recent years, Illumina has faced growing competition from Chinese manufacturers such as MGI, GeneMind, and Cygnus Biosciences. Moreover, the Russian company Syntol has recently developed and commercialized the Nanophor SPS sequencer (Kurochkin et al., 2021). Most contemporary NGS platforms utilize fluorescence-based sequencing-by-synthesis technology within flow cells (Zubov et al., 2021) and share similar library preparation protocols, often with compatible reagents. The library preparation process typically involves several key steps: fragmentation of nucleic acids template (e. g., via ultrasonication or restriction enzyme digestion), ligation of platform-specific short oligonucleotide adapters to one or both ends of the fragments, and amplification of these fragments (Kutnjak et al., 2021). Adapters vary depending on the NGS platform and are required to initiate the sequencing process (Lebas et al., 2022). It is worth noting that second-generation sequencers can only read DNA molecules, therefore, if the template is RNA, fragmentation must be followed by reverse transcription to synthesize cDNA.

Third-generation sequencing (TSG), also known as single-molecule sequencing, is based on reading the sequences of individual molecules and does not require their fragmentation and amplification (Villamor et al., 2019). The maximum read length in this case is several hundred thousand nucleotides, which allows for the complete sequencing of small genomes, such as those of viruses, in a single read (Liu et al., 2025). Important advantages of TGS include the direct sequencing of RNA without reverse transcription to cDNA and the capacity for real-time sequencing (Sun et al., 2022).

Leading manufacturers of third-generation platforms are Pacific Biosciences (USA) and Oxford Nanopore Technologies (UK). Although both technologies allow to eliminate amplification-induced polymerase errors, they have relatively low read quality compared to NGS (Rose et al., 2016; Rang et al., 2018). However, the recently introduced PacBio HiFi technology provides read accuracy exceeding 99.5 % (Han et al., 2024). Nanopore sequencing, originally developed by Oxford Nanopore Technologies, has seen continuous improvements in accuracy and a significant reduction in cost, enhancing its accessibility (Javaran et al., 2021). The Chinese company Qitan Tech has also mastered this technology (Wang et al., 2022). It can be expected that in the future single-molecule sequencing will replace NGS.

## Other factors affecting the quality of plant virome sequencing

As discussed above, the selection of both nucleic acid type (e. g., total RNA, siRNA, dsRNA) and sequencing platform plays a crucial role in plant virome analysis. Sequencing quality and read amount are also affected by a number of other factors.

Contamination is one a major negative factor in any metagenomic analysis (Piombo et al., 2021; Lebas et al., 2022). Sample contamination can occur at all stages, from plant tissue collection to the sequencing process itself (Fig. 1). To monitor contamination during nucleic acid extraction and library preparation, negative controls without template addition are recommended (Fitzpatrick et al., 2021). It is also essential to maintain physical separation between laboratory workflows, use sterile reagents, and ensure the cleanliness of glassware, equipment, and work surfaces (Kutnjak et al., 2021; Maina et al., 2024). Comprehensive guidance for preventing contamination using HTS in plant pathogen diagnostics is provided by S. Massart et al. (2022).

In addition to contamination, virome sequencing quality is strongly influenced by the choice of methods, reagents (particularly enzymes), and equipment. For example, library preparation for second-generation sequencing requires the use of reverse transcriptase and DNA polymerase, both of which can introduce errors during amplification (Cholet et al., 2020). Therefore, high-precision enzymes should be used to minimize artifacts.

The accuracy of virus identification is affected by the number of reads in the dataset. If the read amount is insufficient, viruses present at low titers may remain undetected. Conversely, excessive sequencing increases the risk of false positive identifications (Massart et al., 2014). To determine an optimal sequencing depth, it may be useful to review published virome analyses for the plant species of interest.

## Bioinformatics analysis

Bioinformatics analysis of plant virome sequencing data is a multi-step process that can be performed on both personal computers and high-performance clusters. Difference in computing power affects the speed of analysis. Sequencing reads are processed by specialized tools in an order optimized for the characteristics of a given dataset.

Most bioinformatics tools are developed for Linux operating systems and lack a graphical user interface, requiring command-line use. For other operating systems, cross-platform applications, web-based tools, and commercial software packages are available. The latter are particularly convenient for novice users, as they allow nearly all steps of virome analysis to be performed in a single environment with an intuitive interface. Popular examples include Geneious Prime (New Zealand) and CLC Genomics Workbench (USA). Despite their convenience, the functionality of commercial solutions is limited by the set of built-in tools. While this is generally sufficient for routine virome analysis, the Linux command line offers broader capabilities

Programs designed for Linux systems are typically published as open-source software and are available free of charge. Windows users can rely on the Windows Subsystem for Linux (WSL) or a virtual machine such as VirtualBox. Automated software suites for virus and phytopathogen identification include VirusDetect (Zheng et al., 2017), Virtool (Rott et al., 2017), Kodoja (Baizan-Edge et al., 2019), PhytoPipe (Hu et al., 2023), etc. Such solutions provide rapid results but may not account for dataset-specific characteristics. Better results can be achieved by using programming skills to write your own scripts. In biological data analysis, the most commonly used programming languages are Python and R.

Sequencing results are most often provided as FastQ files containing reads with associated quality scores. For visualizing this information, there is a free program, FastQC (https://www.bioinformatics.babraham.ac.uk/projects/ fastqc), that generates detailed reports.

Regardless of the nucleic acid type or sequencing platform, an important step in virome bioinformatics analysis is preprocessing – a set of procedures designed to improve data quality. Inadequate preprocessing can lead to errors during viral genome assembly and hinder read mapping, ultimately distorting analysis results

Preprocessing typically includes the following steps: – Read filtering. This step involves trimming and removing low-quality and excessively short reads. The user specifies the required quality threshold and minimum read length. For Illumina data, commonly used thresholds include Q20 and Q30, corresponding to read accuracies of 99 and 99.9 %, respectively (Kutnjak et al., 2021). Filtering settings that are too strict can lead to partial loss of informative data, while those that are too lenient can lead to errors during genome mapping and assembly. Filtering also includes the removal of adapter sequences added during library preparation. These operations can be performed using tools such as BBDuk (https:// sourceforge.net/projects/bbmap), Trimmomatic (Bolger et al., 2014), etc. – Merging paired reads based on the overlapping region. This step is only required for paired-end sequencing. – Removing duplicate reads to reduce data volume and increase analysis speed. – Removing reads mapping to the host genome. This step can significantly reduce data volume and improve data quality. However, it also removes viral sequences integrated into the host genome (Pappas et al., 2021). Moreover, the host plant genome assembly may contain errors, including misannotated viral fragments. Thus, host read removal should be applied with caution.

Virus identification in sequencing data relies on comparison of sequences with available databases (Lebas et al., 2022). Identification quality depends directly on database completeness and annotation accuracy. As the database is updated with new sequences, any dataset can be reassessed

**Fig. 2.**
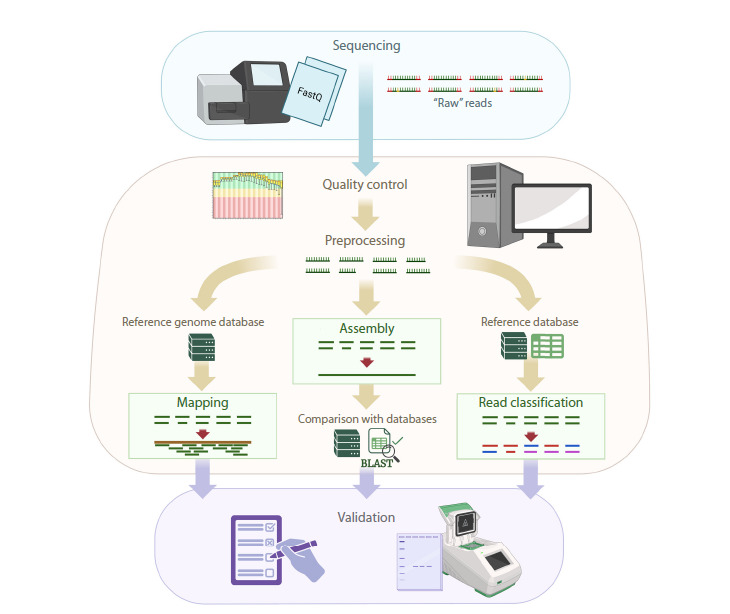
A workflow for plant virome bioinformatics analysis.

There are three main approaches that can complement each other (Fig. 2): read mapping to viral reference sequences; assembly and annotation of contigs; and taxonomic classification of reads without prior assembly

The fastest and simplest approach to virus identification in HTS data is read mapping to viral reference sequences. Mapping is one of the basic operations in the analysis of second-generation sequencing results. The algorithm aligns reads to a reference sequence according to a specified identity threshold. Reads not meeting the threshold are discarded, while the remaining reads align to positions that match the reference sequence

To detect plant viruses using this approach, it is necessary to first prepare a database of reference sequences, which should contain at least one complete genome of each virus capable of infecting the target plant.

Preprocessed reads are mapped to these reference sequences, producing information about coverage, identity levels, and other metrics (Lebas et al., 2022). With sufficient coverage, the mapped reads form a continuous sequence along the entire length of the reference genome

Mapping is usually performed using tools such as BWA (Li, Durbin, 2009), Bowtie2 (Langmead, Salzberg, 2012), Minimap2 (Li, 2018), BBmap (https://sourceforge. net/projects/bbmap), STAR (Dobin, Gingeras, 2015), GraphMap (Sović et al., 2016), and Hisat2 (Kim et al., 2019). The settings and thresholds are very important. Viral genomes can differ markedly from available references due to their rapid mutation rates. If mapping settings are too strict, only isolates closely related to the reference sequence will be detected (Kutnjak et al., 2021). On the other hand, overly permissive settings may allow reads from one virus species to map to another, leading to false positives (Roy et al., 2018).

Mapping enables efficient detection of viruses even when viral read abundance is low. However, results should be interpreted cautiously, considering possible contamination during sample preparation and sequencing. For this reason, threshold values for read count or reference coverage should be established to determine when a sample is considered virus-negative (Belkina et al., 2023).

A second approach to virus identification using HTS is based on de novo assembly of sequences from overlapping reads – contigs – and their comparison with annotated databases. Assembly enables the discovery of novel virus species and new hosts for known viruses, as well as the characterization of genomic variants. Furthermore, this approach eliminates the risk of false negatives associated with a possible incompleteness of the database of reference genomes for viruses of the target plant

Contig assembly can be performed using various algorithms, each with distinct advantages and limitations (White et al., 2017). With sufficient sequencing depth, contigs may correspond to nearly complete viral genomes, excluding short 5′ and 3′ end regions. The efficiency of assembly by the same algorithm varies across different datasets, therefore, it is impossible to choose a single universal one (Sutton et al., 2019; Shvets et al., 2022). Assembly quality can be positively affected by pre-removal of host plant reads and optimization of tool settings (Kutnjak et al., 2021).

Common assemblers for short reads include SPAdes (Bankevich et al., 2012), Velvet (Zerbino, Birney, 2008), Geneious (https://www. geneious.com), CLC (https:// digitalinsights.qiagen.com), MIRA (Chevreux et al., 1999), ABySS (Simpson et al., 2009), IDBA-UI (Peng et al., 2012), SOAPdenovo2 (Luo et al., 2012); for long reads, Canu (Koren et al., 2017), Falcon (Chin et al., 2016), and Pomoxis (https://github.com/nanoporetech/pomoxis). Some algorithms, such as SPAdes and Unicycler (Wick et al., 2017), perform well with hybrid datasets combining short and long reads (Pappas et al., 2021).

To increase reliability, it is recommended to use several assembly algorithms. Note that depending on the library size and the chosen algorithm, the assembly may take a long time and require a lot of RAM.

After assembly, contigs are compared against annotated sequence databases. Result reliability depends largely on database completeness and quality. Many of them are available online, but it is often more convenient to deploy the database on a cloud server or locally when storage capacity allows

The most convenient, up-to-date, and frequently used databases are maintained by NCBI; by early 2025, they contained approximately six million nucleotide sequences and one million amino acid sequences (Sayers et al., 2025). NCBI provides both the curated RefSeq database, which includes one genome per organism (https://www.ncbi.nlm. nih.gov/refseq), and the community-updated GenBank database, which contains the majority of publicly available sequences (https://www.ncbi.nlm.nih.gov/genbank). However, GenBank entries often lack thorough quality control or annotation verification. Thus, comparisons of contigs against the GenBank database can lead to misidentifications. Using the database of reference virus genomes, Viral RefSeq, will potentially yield the most reliable results, but its updates occur slowly and may lack a significant number of recently discovered viruses.

The most widely used tool for comparing contigs against databases is BLAST, which integrates several algorithms for analyzing nucleotide and amino acid sequences. The fastest algorithm that can be used to determine which virus in the database corresponds to a particular contig is megablast. For discovering novel viruses, blastx or tblastx are preferred due to their ability to detect distant sequence similarities. The main drawback of blastx and especially tblastx is the low speed of analysis; therefore, when time is limited, the faster DIAMOND algorithm can be used (Buchfink et al., 2015). However, some researchers believe that it is less effective for virus identification (Kutnjak et al., 2021).

BLAST can be run via the NCBI website (https://blast. ncbi.nlm.nih.gov/Blast.cgi), which is convenient for a small number of sequences and requires no local computing resources. BLAST is also integrated into graphical environments such as Geneious Prime and CLC Genomics Workbench. For command-line analysis in Linux, macOS, and Windows, the BLAST+ suite is used (Camacho et al., 2009).

As a result of comparison with the virus sequence database, contigs are assigned to specific species with varying reliability. The level of reliability is typically indicated using the E-value, which reflects the probability of a random match between a contig and a database entry (VanderWeele, Ding, 2017). The closer the E-value to 0, the more reliable the identification Approaches based on taxonomic classification of reads without prior assembly or mapping are less common in plant virome studies. However, if the necessary computing resources are available, they can produce results much faster than the approaches discussed above. Popular taxonomic read classifiers include Kraken2 (Wood et al., 2019), Kaiju (Menzel et al., 2016), CLARK-S (Ounit, Lonardi, 2016), and Centrifuge (Kim et al., 2016). These tools typically require large databases, substantial computing power, and proficiency with the Linux command line.

## Validation and interpretation of virome analysis

After detecting reads from a particular virus in a library using any bioinformatics method, the results must be interpreted correctly. Because sequencing quality depends on many factors, no universal criteria exist for determining how many reads or contigs reliably indicate infection (Villamor et al., 2019). Moreover, analyzing the same dataset using different software or different settings may lead to different results.

Therefore, each case requires a comprehensive and critical evaluation of the data. During the final steps of virome analysis, the researcher’s expertise and biological understanding become crucial. In particular, the researcher must understand that not all viruses detected in the samples are pathogens of the sampled plant. Virome data may include viruses of insects, fungi, and bacteria associated with the plant, as well as human or animal viruses (Cobbin et al., 2021). This is particularly important to remember when results appear to indicate the discovery of previously undescribed viruses.

To reliably identify plant viruses in metagenomic data, bioinformatics analysis results should be confirmed by molecular genetic methods such as PCR or reverse transcription- PCR (RT-PCR), depending on the viral genome type. When a virus is detected in a sample both by virome sequencing and by molecular diagnostics, identification can be considered highly reliable.

## Conclusion

Virome analysis using high-throughput sequencing is a powerful method that supports both fundamental and applied research in agriculture. Each year, high-throughput sequencing becomes more accessible as analytical and instrumental capabilities expand. In the future, once an appropriate regulatory framework is established, this method may form the basis for decision-making in national food security strategies.

## Conflict of interest

The authors declare no conflict of interest.

## References

Atallah S.S., Gómez M.I., Fuchs M.F., Martinson T.E. Economic impact
of grapevine leafroll disease on Vitis vinifera cv. Cabernet Franc in
Finger Lakes vineyards of New York. Am J Enol Vitic. 2012;63(1):
73-79. doi 10.5344/ajev.2011.11055

Baizan-Edge A., Cock P., MacFarlane S., McGavin W., Torrance L.,
Jones S. Kodoja: a workflow for virus detection in plants using k-mer
analysis of RNA-sequencing data. J Gen Virol. 2019;100(3):533-
542. doi 10.1099/jgv.0.001210

Bankevich A., Nurk S., Antipov D., Gurevich A.A., Dvorkin M., Kulikov
A.S., Lesin V.M., … Sirotkin A.V., Vyahhi N., Tesler G., Alekseyev
M.A., Pevzner P.A. SPAdes: a new genome assembly algorithm
and its applications to single-cell sequencing. J Comput Biol.
2012;19(5):455-477. doi 10.1089/cmb.2012.0021

Belkina D., Karpova D., Porotikova E., Lifanov I., Vinogradova S.
Grapevine virome of the Don ampelographic collection in Russia
has concealed five novel viruses. Viruses. 2023;15(12):2429. doi
10.3390/v15122429

Bolger A.M., Lohse M., Usadel B. Trimmomatic: a flexible trimmer for
Illumina sequence data. Bioinformatics. 2014;30(15):2114-2120. doi
10.1093/bioinformatics/btu170

Buchfink B., Xie C., Huson D.H. Fast and sensitive protein alignment
using DIAMOND. Nat Methods. 2015;12(1):59-60. doi 10.1038/
nmeth.3176

Camacho C., Coulouris G., Avagyan V., Ma N., Papadopoulos J., Bealer
K., Madden T.L. BLAST+: architecture and applications. BMC
Bioinformatics. 2009;10(1):421. doi 10.1186/1471-2105-10-421

Chevreux B., Wetter T., Suhai S. Genome sequence assembly using
trace signals and additional sequence information. In: Proceedings
of the German Conference on Bioinformatics (GCB) 99. Hannover,
1999;45-56

Chin C.-S., Peluso P., Sedlazeck F.J., Nattestad M., Concepcion G.T.,
Clum A., Dunn C., … Luo C., Ecker J.R., Cantu D., Rank D.R.,
Schatz M.C. Phased diploid genome assembly with single-molecule
real-time sequencing. Nat Methods. 2016;13(12):1050-1054. doi
10.1038/nmeth.4035

Cholet F., Ijaz U.Z., Smith C.J. Reverse transcriptase enzyme and priming
strategy affect quantification and diversity of environmental
transcripts. Environ Microbiol. 2020;22(6):2383-2402. doi 10.1111/
1462-2920.15017

Cobbin J.C., Charon J., Harvey E., Holmes E.C., Mahar J.E. Current
challenges to virus discovery by meta-transcriptomics. Curr Opin
Virol. 2021;51:48-55. doi 10.1016/j.coviro.2021.09.007

Crossley B.M., Bai J., Glaser A., Maes R., Porter E., Killian M.L.,
Clement T., Toohey-Kurth K. Guidelines for Sanger sequencing and
molecular assay monitoring. J Vet Diagn Invest. 2020;32(6):767-
775. doi 10.1177/1040638720905833

Dobin A., Gingeras T.R. Mapping RNA‐seq reads with STAR. Curr
Protoc Bioinformatics. 2015;51(1):11.14.1-11.14.19. doi 10.1002/
0471250953.bi1114s51

Fall M.L., Xu D., Lemoyne P., Clément G., Moffett P., Ritzenthaler C.
An innovative binding‐protein‐based dsRNA extraction method:
comparison of cost‐effectiveness of virus detection methods using
high‐throughput sequencing. Mol Ecol Resour. 2025;25(7):e14111.
doi 10.1111/1755-0998.14111

Filloux D., Dallot S., Delaunay A., Galzi S., Jacquot E., Roumagnac P.
Metagenomics approaches based on virion-associated nucleic acids
(VANA): an innovative tool for assessing without a priori viral diversity
of plants. In: Lacomme C. (Ed.) Plant Pathology. Methods
in Molecular Biology. Vol. 1302. New York: Humana Press, 2015;
249-257. doi 10.1007/978-1-4939-2620-6_18

Fitzpatrick A.H., Rupnik A., O’Shea H., Crispie F., Keaveney S., Cotter
P. High throughput sequencing for the detection and characterization
of RNA viruses. Front Microbiol. 2021;12:621719. doi 10.3389/
fmicb.2021.621719

Gaafar Y.Z.A., Ziebell H. Comparative study on three viral enrichment
approaches based on RNA extraction for plant virus/viroid detection
using high-throughput sequencing. PLoS One. 2020;15(8):
e0237951. doi 10.1371/journal.pone.0237951Gallo Y., Marín M., Gutiérrez P. Detection of RNA viruses in Solanum
quitoense by high-throughput sequencing (HTS) using total and
double stranded RNA inputs. Physiol Mol Plant Pathol. 2021;113:
101570. doi 10.1016/j.pmpp.2020.101570

Han Y., He J., Li M., Peng Y., Jiang H., Zhao J., Li Y., Deng F. Unlocking
the potential of metagenomics with the PacBio high-fidelity
sequencing technology. Microorganisms. 2024;12(12):2482. doi
10.3390/microorganisms12122482

Hu X., Hurtado-Gonzales O.P., Adhikari B.N., French-Monar R.D.,
Malapi M., Foster J.A., McFarland C.D. PhytoPipe: a phytosanitary
pipeline for plant pathogen detection and diagnosis using RNA-seq
data. BMC Bioinformatics. 2023;24(1):470. doi 10.1186/s12859-
023-05589-2

Javaran V.J., Moffett P., Lemoyne P., Xu D., Adkar-Purushothama C.R.,
Fall M.L. Grapevine virology in the third-generation sequencing
era: from virus detection to viral epitranscriptomics. Plants. 2021;
10(11):2355. doi 10.3390/plants10112355

Jones R.A.C. Global plant virus disease pandemics and epidemics.
Plants. 2021;10(2):233. doi 10.3390/plants10020233

Kim D., Song L., Breitwieser F.P., Salzberg S.L. Centrifuge: rapid and
sensitive classification of metagenomic sequences. Genome Res.
2016;26(12):1721-1729. doi 10.1101/gr.210641.116

Kim D., Paggi J.M., Park C., Bennett C., Salzberg S.L. Graph-based
genome alignment and genotyping with HISAT2 and HISAT-genotype.
Nat Biotechnol. 2019;37(8):907-915. doi 10.1038/s41587-
019-0201-4

Koren S., Walenz B.P., Berlin K., Miller J.R., Bergman N.H., Phillippy
A.M. Canu: scalable and accurate long-read assembly via
adaptive
k-mer weighting and repeat separation. Genome Res. 2017;
27(5):722-736. doi 10.1101/gr.215087.116

Kurochkin V.E., Alekseev Y.I., Petrov D.G., Evstrapov A.A. Domestic
devices for molecular genetic analysis: developments of the
IAP RAS and SINTOL LLC. Russ Mil Med Acad Rep. 2021;40(3):
69-74. doi 10.17816/rmmar76918 (in Russian)

Kutnjak D., Tamisier L., Adams I., Boonham N., Candresse T., Chiumenti
M., De Jonghe K., … Rollin J., Rott M., Schumpp O., Massart
S., Haegeman A. A primer on the analysis of high-throughput
sequencing data for detection of plant viruses. Microorganisms.
2021;9(4):841. doi 10.3390/microorganisms9040841

Langmead B., Salzberg S.L. Fast gapped-read alignment with Bowtie 2.
Nat Methods. 2012;9(4):357-359. doi 10.1038/nmeth.1923

Lebas B., Adams I., Al Rwahnih M., Baeyen S., Bilodeau G.J.,
Blouin
A.G., Boonham N., … Vicente C.S.L., Vossenberg B.T.L.H.,
Wetzel T., Ziebell H., Massart S. Facilitating the adoption of highthroughput
sequencing technologies as a plant pest diagnostic test
in laboratories: a step‐by‐step description. EPPO Bull. 2022;52(2):
394-418. doi 10.1111/epp.12863

Lee H.-K., Kim S.-Y., Yang H.-J., Lee D.-S., Kwon B., Lee D.-Y., Oh J.,
Lee S.-H. The detection of plant viruses in Korean ginseng (Panax
ginseng) through RNA sequencing. Plant Pathol J. 2020;36(6):
643-650. doi 10.5423/PPJ.NT.07.2020.0137

Lefeuvre P., Martin D.P., Elena S.F., Shepherd D.N., Roumagnac P.,
Varsani A. Evolution and ecology of plant viruses. Nat Rev Microbiol.
2019;17(10):632-644. doi 10.1038/s41579-019-0232-3

Li H. Minimap2: pairwise alignment for nucleotide sequences. Bioinformatics.
2018;34(18):3094-3100. doi 10.1093/bioinformatics/
bty191

Li H., Durbin R. Fast and accurate short read alignment with Burrows–
Wheeler transform. Bioinformatics. 2009;25(14):1754-1760. doi
10.1093/bioinformatics/btp324

Liu S., Rodriguez J.S., Munteanu V., Ronkowski C., Sharma N.K., Alser
M., Andreace F., … Ganda E., Davenport E.R., Pop M., Koslicki
D., Mangul S. Analysis of metagenomic data. Nat Rev Methods
Primers. 2025;5(1):5. doi 10.1038/s43586-024-00376-6

Luo R., Liu B., Xie Y., Li Z., Huang W., Yuan J., He G., … Li Y.,
Yang H., Wang J., Lam T.-W., Wang J. SOAPdenovo2: an empirically
improved memory-efficient short-read de novo assembler.
GigaScience.
2012;1(1):18. doi 10.1186/2047-217X-1-18

Maina S., Donovan N.J., Plett K., Bogema D., Rodoni B.C. Highthroughput
sequencing for plant virology diagnostics and its potential
in plant health certification. Front Hortic. 2024;3:1388028. doi
10.3389/fhort.2024.1388028

Maliogka V.I., Minafra A., Saldarelli P., Ruiz-García A.B., Glasa M.,
Katis N., Olmos A. Recent advances on detection and characterization
of fruit tree viruses using high-throughput sequencing technologies.
Viruses. 2018;10(8):436. doi 10.3390/v10080436

Massart S., Olmos A., Jijakli H., Candresse T. Current impact and future
directions of high throughput sequencing in plant virus diagnostics.
Virus Res. 2014;188:90-96. doi 10.1016/j.virusres.2014.
03.029

Massart S., Adams I., Al Rwahnih M., Baeyen S., Bilodeau G.J.,
Blouin
A.G., Boonham N., … van de Vossenberg B.T.L.H., Westenberg
M., Wetzel T., Ziebell H., Lebas B.S.M. Guidelines for the reliable
use of high throughput sequencing technologies to detect plant
pathogens and pests. Peer Commun J. 2022;2:e62. doi 10.24072/
pcjournal.181

Menzel P., Ng K.L., Krogh A. Fast and sensitive taxonomic classification
for metagenomics with Kaiju. Nat Commun. 2016;7(1):11257.
doi 10.1038/ncomms11257

Moubset O., François S., Maclot F., Palanga E., Julian C., Claude L.,
Fernandez E., … Massart S., Ogliastro M., Martin D.P., Filloux D.,
Roumagnac P. Virion-associated nucleic acid-based metagenomics:
a decade of advances in molecular characterization of plant viruses.
Phytopathology. 2022;112(11):2253-2272. doi 10.1094/PHYTO-03-
22-0096-RVW

Nabeshima T., Abe J. High-throughput sequencing indicates novel
Varicosavirus, Emaravirus, and Deltapartitivirus infections in Vitis
coignetiae. Viruses. 2021;13(5):827. doi 10.3390/v13050827

Ounit R., Lonardi S. Higher classification sensitivity of short metagenomic
reads with CLARK-S. Bioinformatics. 2016;32(24):3823-
3825. doi 10.1093/bioinformatics/btw542

Panno S., Davino S., Caruso A.G., Bertacca S., Crnogorac A., Mandić A.,
Noris E., Matić S. A review of the most common and economically
important diseases that undermine the cultivation of tomato crop in
the mediterranean basin. Agronomy. 2021;11(11):2188. doi 10.3390/
agronomy11112188

Pappas N., Roux S., Hölzer M., Lamkiewicz K., Mock F., Marz M.,
Dutilh B.E. Virus bioinformatics. In: Encyclopedia of Virology. Elsevier,
2021;124-132. doi 10.1016/B978-0-12-814515-9.00034-5

Pecman A., Kutnjak D., Gutiérrez-Aguirre I., Adams I., Fox A., Boonham
N., Ravnikar M. Next generation sequencing for detection
and discovery of plant viruses and viroids: comparison of two approaches.
Front Microbiol. 2017;8:1998. doi 10.3389/fmicb.2017.
01998

Peng Y., Leung H.C.M., Yiu S.M., Chin F.Y.L. IDBA-UD: a de novo
assembler for single-cell and metagenomic sequencing data with
highly uneven depth. Bioinformatics. 2012;28(11):1420-1428. doi
10.1093/bioinformatics/bts174

Piombo E., Abdelfattah A., Droby S., Wisniewski M., Spadaro D.,
Schena L. Metagenomics approaches for the detection and surveillance
of emerging and recurrent plant pathogens. Microorganisms.
2021;9(1):188. doi 10.3390/microorganisms9010188

Rang F.J., Kloosterman W.P., de Ridder J. From squiggle to basepair:
computational approaches for improving nanopore sequencing read
accuracy. Genome Biol. 2018;19(1):90. doi 10.1186/s13059-018-
1462-9

Roossinck M.J., Martin D.P., Roumagnac P. Plant virus metagenomics:
advances in virus discovery. Phytopathology. 2015;105(6):716-727.
doi 10.1094/PHYTO-12-14-0356-RVW

Rose R., Constantinides B., Tapinos A., Robertson D.L., Prosperi M.
Challenges in the analysis of viral metagenomes. Virus Evol. 2016;
2(2):vew022. doi 10.1093/ve/vew022

Rott M., Xiang Y., Boyes I., Belton M., Saeed H., Kesanakurti P.,
Hayes S., Lawrence T., Birch C., Bhagwat B., Rast H. Application of
next generation sequencing for diagnostic testing of tree fruit viruses
and viroids. Plant Dis. 2017;101(8):1489-1499. doi 10.1094/PDIS-
03-17-0306-RE

Roy S., Coldren C., Karunamurthy A., Kip N.S., Klee E.W., Lincoln
S.E., Leon A., Pullambhatla M., Temple-Smolkin R.L., Voelkerding
K.V., Wang C., Carter A.B. Standards and guidelines for validating
next-generation sequencing bioinformatics pipelines. J Mol
Diagn. 2018;20(1):4-27. doi 10.1016/j.jmoldx.2017.11.003

Rubino L., Abrahamian P., An W., Aranda M.A., Ascencio-Ibañez J.T.,
Bejerman N., Blouin A.G., … Whitfield A.E., Wylie S.J., Yang C.,
Zerbini F.M., Zhang S. Summary of taxonomy changes ratified by
the International Committee on Taxonomy of Viruses from the Plant
Viruses Subcommittee, 2025. J Gen Virol. 2025;106(7):002114. doi
10.1099/jgv.0.002114

Sayers E.W., Beck J., Bolton E.E., Brister J.R., Chan J., Connor R.,
Feldgarden M., … Wang J., Ye J., Zellers E., Schneider V.A.,
Pruitt K.D. Database resources of the National Center for Biotechnology
Information in 2025. Nucleic Acids Res. 2025;53(D1):D20-
D29. doi 10.1093/nar/gkae979

Shvets D., Sandomirsky K., Porotikova E., Vinogradova S. Metagenomic
analysis of ampelographic collections of Dagestan revealed
the presence of two novel grapevine viruses. Viruses. 2022;14(12):
2623. doi 10.3390/v14122623

Simpson J.T., Wong K., Jackman S.D., Schein J.E., Jones S.J.M., Birol
İ. ABySS: a parallel assembler for short read sequence data.
Genome
Res. 2009;19(6):1117-1123. doi 10.1101/gr.089532.108

Sović I., Šikić M., Wilm A., Fenlon S.N., Chen S., Nagarajan N. Fast and
sensitive mapping of nanopore sequencing reads with GraphMap.
Nat Commun. 2016;7(1):11307. doi 10.1038/ncomms11307

Sun K., Liu Y., Zhou X., Yin C., Zhang P., Yang Q., Mao L., Shentu X.,
Yu X. Nanopore sequencing technology and its application in plant
virus diagnostics. Front Microbiol. 2022;13:939666. doi 10.3389/
fmicb.2022.939666

Sutton T.D.S., Clooney A.G., Ryan F.J., Ross R.P., Hill C. Choice of
assembly software has a critical impact on virome characterisation.
Microbiome. 2019;7(1):12. doi 10.1186/s40168-019-0626-5

Turco S., Golyaev V., Seguin J., Gilli C., Farinelli L., Boller T.,
Schumpp O., Pooggin M.M. Small RNA-omics for virome reconstruction
and antiviral defense characterization in mixed infections
of cultivated Solanum plants. Mol Plant Microbe Interact. 2018;
31(7):707-723. doi 10.1094/MPMI-12-17-0301-R

VanderWeele T.J., Ding P. Sensitivity analysis in observational research:
introducing the E-value. Ann Intern Med. 2017;167(4):268-274. doi
10.7326/M16-2607

Villamor D.E.V., Ho T., Al Rwahnih M., Martin R.R., Tzanetakis I.E.
High throughput sequencing for plant virus detection and discovery.
Phytopathology. 2019;109(5):716-725. doi 10.1094/PHYTO-07-18-
0257-RVW

Vinogradova S., Porotikova E., Navrotskaya E., Galbacs Z.N., Massart
S., Varallyay E. The first virome of a Russian vineyard. Plants.
2023;12(18):3292. doi 10.3390/plants12183292

Vivek A.T., Zahra S., Kumar S. From current knowledge to best practice:
a primer on viral diagnostics using deep sequencing of virusderived
small interfering RNAs (vsiRNAs) in infected plants.
Methods. 2020;183:30-37. doi 10.1016/j.ymeth.2019.10.009

Wang Z., Qin L., Liu J., Jiang L., Zou X., Chen X., Song F., Dai H.,
Hou Y. Forensic nanopore sequencing of microhaplotype markers
using QitanTech’s QNome. Forensic Sci Int Genet. 2022;57:102657.
doi 10.1016/j.fsigen.2021.102657

White D.J., Wang J., Hall R.J. Assessing the impact of assemblers on virus
detection in a de novo metagenomic analysis pipeline. J Comput
Biol. 2017;24(9):874-881. doi 10.1089/cmb.2017.0008

Wick R.R., Judd L.M., Gorrie C.L., Holt K.E. Unicycler: resolving
bacterial genome assemblies from short and long sequencing reads.
PLoS Comput Biol. 2017;13(6):e1005595. doi 10.1371/journal.pcbi.
1005595

Wood D.E., Lu J., Langmead B. Improved metagenomic analysis with
Kraken 2. Genome Biol. 2019;20(1):257. doi 10.1186/s13059-019-
1891-0

Zerbino D.R., Birney E. Velvet: algorithms for de novo short read assembly
using de Bruijn graphs. Genome Res. 2008;18(5):821-829.
doi 10.1101/gr.074492.107

Zheng Y., Gao S., Padmanabhan C., Li R., Galvez M., Gutierrez D.,
Fuentes S., Ling K.-S., Kreuze J., Fei Z. VirusDetect: an automated
pipeline for efficient virus discovery using deep sequencing of
small RNAs. Virology. 2017;500:130-138. doi 10.1016/j.virol.2016.
10.017

Zhuravlyov V.S., Dolgikh V.V., Timofeev S.A., Gannibal F.B. RNA
interference method in plant protection against insect pests. Plant
Prot News. 2022;105(1):28-39. doi 10.31993/2308-6459-2022-105-
1-15219

Zubov V.V., Chemeris D.A., Vasilov R.G., Kurochkin V.E., Alekseev
Ya.I. Brief history of high-throughput nucleic acid sequencing
methods. Biomics. 2021;13(1):27-46. doi 10.31301/2221-6197.
bmcs.2021-4

